# The *Drosophila* Pericentrin-like-protein (PLP) cooperates with Cnn to maintain the integrity of the outer PCM

**DOI:** 10.1242/bio.012914

**Published:** 2015-07-08

**Authors:** Jennifer H. Richens, Teresa P. Barros, Eliana P. Lucas, Nina Peel, David Miguel Susano Pinto, Alan Wainman, Jordan W. Raff

**Affiliations:** 1 Sir William Dunn School of Pathology, University of Oxford, South Parks Rd, Oxford OX1 3RE, UK; 2 The Gurdon Institute, University of Cambridge, Tennis Court Rd, Cambridge CB2 1QN, UK; 3 Micron Oxford Advanced Bioimaging Unit, Department of Biochemistry, University of Oxford, South Parks Rd, Oxford OX1 3QU, UK

**Keywords:** Centriole, Centrosome, Mitosis, PCM, Pericentrin, Spindle

## Abstract

Centrosomes comprise a pair of centrioles surrounded by a matrix of pericentriolar material (PCM). In vertebrate cells, Pericentrin plays an important part in mitotic PCM assembly, but the *Drosophila* Pericentrin-like protein (PLP) appears to have a more minor role in mitotic fly cells. Here we investigate the function of PLP during the rapid mitotic cycles of the early *Drosophila* embryo. Unexpectedly, we find that PLP is specifically enriched in the outer-most regions of the PCM, where it largely co-localizes with the PCM scaffold protein Cnn. In the absence of PLP the outer PCM appears to be structurally weakened, and it rapidly disperses along the centrosomal microtubules (MTs). As a result, centrosomal MTs are subtly disorganized in embryos lacking PLP, although mitosis is largely unperturbed and these embryos develop and hatch at near-normal rates. Y2H analysis reveals that PLP can potentially form multiple interactions with itself and with the PCM recruiting proteins Asl, Spd-2 and Cnn. A deletion analysis suggests that PLP participates in a complex network of interactions that ultimately help to strengthen the PCM.

## INTRODUCTION

Centrosomes are the major microtubule (MT) organizing centers (MTOCs) in many eukaryotic cells and they play an important part in many cell processes, including establishing and maintaining cell polarity and cell division (Bornens, 2012; Nigg and Raff, 2009). Centrosomes are formed when centrioles recruit a matrix of pericentriolar material (PCM) around themselves, and the PCM probably comprises several hundred proteins (Alves-Cruzeiro et al., 2013; Andersen et al., 2003; Müller et al., 2010). These include proteins that nucleate and organize MTs as well as many cell cycle regulators, checkpoint proteins and cell signaling molecules (Doxsey et al., 2005; Mennella et al., 2013). In most cell types the centrioles organize relatively small amounts of PCM during interphase, but the PCM expands dramatically as cells prepare to enter mitosis – a process termed centrosome maturation (Mahen and Venkitaraman, 2012; Palazzo et al., 2000; Woodruff et al., 2014).

Recent studies have indicated that centrioles may use different mechanisms to organize the PCM in interphase and in mitosis. In interphase, the mother centriole appears to recruit a small amount of PCM that is highly organized (Fu and Glover, 2012; Lawo et al., 2012; Mennella et al., 2012; Sonnen et al., 2012): in particular the centriole and PCM protein Pericentrin – the Pericentrin-like-protein (PLP or CP309) in flies (Kawaguchi and Zheng, 2004; Martinez-Campos et al., 2004) – exhibits a stereotypical organization, with its C-terminal PACT domain in close contact with the mother centriole and its N-terminus extending outwards away from the centriole. Most other PCM components lie within the region bounded by the extended Pericentrin/PLP molecules and, in fly cultured cells, the depletion of PLP severely disrupts the recruitment of the other interphase PCM components (Mennella et al., 2012). In contrast, the expanded mitotic PCM has little detectable molecular organization, and although PLP is detectable in the mitotic PCM, no stereotypical organization of PLP, or of the PCM, can be discerned (Fu and Glover, 2012; Lawo et al., 2012; Mennella et al., 2012; Sonnen et al., 2012).

Pericentrin has been strongly implicated in mitotic PCM recruitment in vertebrate cells (Delaval and Doxsey, 2010), where it can interact with other key PCM components such as the γ-tubulin ring complex and Cdk5Rap2/Cep215 (Buchman et al., 2010; Chen et al., 2014; Haren et al., 2009; Zimmerman et al., 2004), and its phosphorylation by Plk1 seems to be essential for mitotic PCM recruitment in at least some cultured cell types (Lee and Rhee, 2011). In somatic fly cells, however, PLP seems to play a more minor role in mitotic PCM recruitment; although the PCM is clearly disorganized during early mitosis in cells lacking PLP, by metaphase the PCM appears largely unperturbed (Martinez-Campos et al., 2004). Instead, recent studies have indicated that in mitotic fly cells the centriole protein Asl (Cep152 in humans) plays an important part in recruiting Spd-2 (Cep192 in humans) and Centrosomin (Cnn–Cdk5Rap2/Cep215 in humans) to mother centrioles, and these proteins then cooperate to form a scaffold structure around the mother centrioles; this scaffold appears to be responsible for recruiting most other PCM proteins to the mitotic centrosome (Conduit et al., 2010, 2014a,b). Interestingly, several studies in vertebrate cells have suggested a link between Pericentrin and Cdk5Rap2/Cep215 function in PCM recruitment (Buchman et al., 2010; Chen et al., 2014; Haren et al., 2009; Kim and Rhee, 2014) and also in maintaining centriole cohesion and regulating centriole disengagement (Barrera et al., 2010; Lee and Rhee, 2012; Pagan et al., 2015). Moreover, mutations in Cdk5Rap2 have been linked to autosomal primary microcephaly (MCPH) while mutations in Pericentrin have been linked to microcephalic osteodysplastic primordial dwarfism (Bond et al., 2005; Griffith et al., 2008; Rauch et al., 2008).

To better understand how PLP might contribute to mitotic centrosome function in flies we decided to study its function in the early *Drosophila* embryo. These embryos undergo a series of rapid nuclear divisions comprising only alternating S- and M-phases and, unlike in most fly somatic cells where centrioles and centrosomes are dispensable for viability (Basto et al., 2006), centrosomes are essential for the viability of the early embryo (Stevens et al., 2007; Varmark et al., 2007). Embryos lacking centrosomes arrest early in development after only a few rounds of nuclear division, and several key PCM proteins such as Cnn, Spd-2, Asl and TACC that are not essential for fly viability are essential for these early stages of embryo development (Dix and Raff, 2007; Gergely et al., 2000; Megraw et al., 1999; Varmark et al., 2007). We therefore reasoned that if PLP had an important function in mitotic centrosome assembly in flies this would most likely be manifested during the rapid nuclear divisions of the early embryo.

Investigating PLP function in early embryos is not trivial, as PLP/Pericentrin is a component of both the centrioles and the centrosome and it is essential for proper cilia function in flies and vertebrate cells (Jurczyk et al., 2004; Martinez-Campos et al., 2004). Flies lacking cilia are uncoordinated as cilia are essential for the function of Type I sensory neurons that are responsible for mechano- and chemo-sensation (Kernan et al., 1994). As a result, PLP mutant flies cannot mate, and die shortly after eclosion. Here we used two independent methods to generate fly embryos that lack endogenous PLP. Our studies reveal that PLP is not essential for early embryo development and that centrosome and MT behavior is only subtly perturbed in the absence of PLP. Unexpectedly, we find that a fraction of PLP is enriched in the outer-region of the PCM and it appears to interact with the Cnn scaffold in this region to strengthen the PCM.

## RESULTS AND DISCUSSION

### PLP is concentrated at centrioles but is also enriched in the outer regions of the PCM

**Fig. 1.**
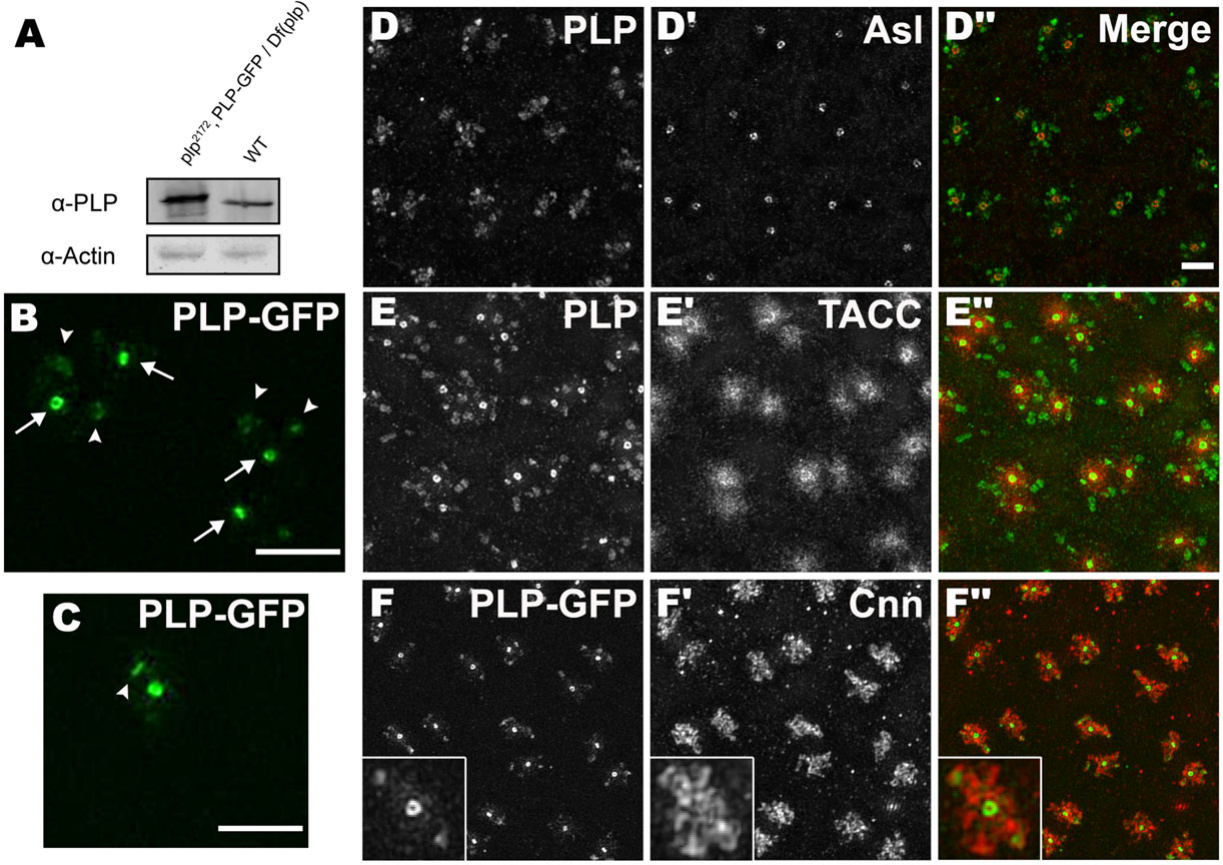
**PLP is concentrated at mother centrioles and in the outer regions of the PCM.** (A) A western blot comparing the expression level of PLP-GFP in *plp* mutant embryos (left lane) to endogenous PLP in WT embryos (right lane). PLP-GFP runs more slowly than endogenous PLP and is overexpressed by ∼2-fold. Actin is shown as a loading control. (B,C) 3D-SIM images from living *plp* mutant embryos expressing PLP-GFP. In B, arrows highlight the position of mother centrioles, arrowheads highlight the concentration of PLP in the outer regions of the PCM. In C, arrowhead highlights the position of a new mother centriole (in side view) that has just separated from the original mother centriole at the end of mitosis; the new mother centriole is just starting to incorporate PLP-GFP. (D-F″) Images from fixed embryos showing the distribution of endogenous PLP (left panels, green in merged images) and the centriole or PCM markers Asl, TACC or Cnn (middle panels, red in merged images), as indicated. Inset in (F-F″) shows a 2× magnified view of a centrosome illustrating the partial overlap of Cnn and PLP in the outer PCM. Scale bar=2 µm.

We previously showed that antibodies raised against PLP predominantly stain centrioles in *Drosophila* somatic cells; a GFP-fusion to the 226 aa C-terminal PACT domain was also strongly concentrated in centrioles, but was also more weakly detectable in the PCM in early embryos (Martinez-Campos et al., 2004). To examine the distribution of full length PLP in more detail, we generated a transgenic line driving the expression of a full length PLP-GFP fusion protein under the control of the Ubq promoter. This protein was overexpressed by ∼2-fold compared to the endogenous protein (Fig. 1A), and it rescued the *plp* mutant phenotype in embryos (see below). We analyzed the behavior of PLP-GFP in living embryos using 3D-structured illumination super-resolution microscopy (3D-SIM) (Fig. 1B,C). As described previously in fixed cells (Fu and Glover, 2012; Mennella et al., 2012), PLP was strongly concentrated around the mother centrioles (arrows, Fig. 1B, arrowhead in Fig. 1C indicates a side on view of a new mother centriole after centriole separation) and was also weakly detectable in the PCM; unexpectedly, however, we observed that PLP-GFP was particularly enriched in certain regions of the outermost PCM (arrowheads, Fig. 1B). A similar distribution was also observed with the endogenous PLP protein in fixed embryos (Fig. 1D,E)

Different PCM proteins exhibit different distributions within the mitotic PCM (Conduit et al., 2014b). In fly embryos, PCM proteins such as Spd-2, DGrip71, Polo and Aurora A are largely concentrated in an inner region of PCM; proteins such as Cnn and TACC extend further outwards into the more peripheral regions; proteins such as Msps (the fly homologue of ch-Tog/XMAP215) and γ-tubulin exhibit a more intermediate distribution (Conduit et al., 2014b) (see below). The outward extension of the peripheral PCM components Cnn and TACC is driven by an interaction with the centrosomal MTs, and both proteins exhibit extensive centrosomal “flaring” – where aggregates of the proteins at the periphery move away from the centrosome on the centrosomal MTs and eventually break away from the bulk of the PCM; the distribution of TACC and Cnn flares partially overlap, but are distinct (Lee et al., 2001; Megraw et al., 2002). We wondered whether the PLP fraction enriched at the periphery of the PCM might be specifically interacting with the peripheral Cnn and/or TACC flares. To test this possibility we compared the distribution of PLP with that of Cnn or TACC in fixed embryos. In 3D-SIM images TACC was rather diffusely detected throughout the PCM volume and showed no obvious enrichment in the outer regions where PLP was concentrated (Fig. 1E). In contrast, the distribution of Cnn and PLP often closely overlapped in the peripheral regions of the PCM, although this overlap was not complete: virtually all the peripheral regions that contained high levels of PLP were closely associated with peripheral regions of the Cnn scaffold, but many peripheral regions of the Cnn scaffold were not obviously associated with high levels of PLP (Fig. 1F).

**Fig. 2.**
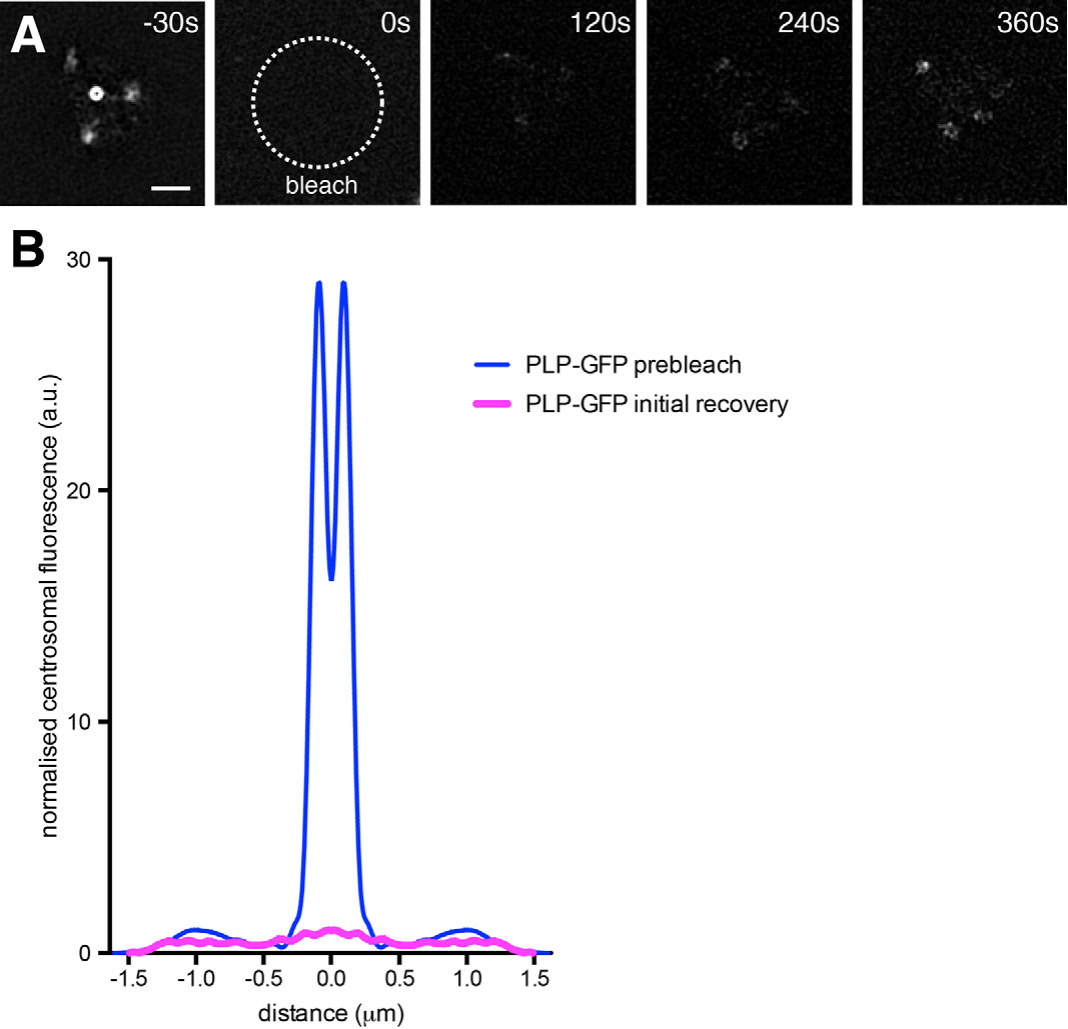
**A 3D-SIM analysis of PLP-GFP protein dynamics.** (A) 3D SIM images show a single centrosome in a living *plp* mutant embryo expressing PLP-GFP that was photobleached at t=0 s. The recovery of the PLP-GFP signal was then monitored over time. Note how PLP-GFP fluorescence recovers in the PCM, and in particular in the outer regions of the PCM that were enriched with PLP-GFP, but does not detectably recover around the central mother centriole. (B) The graph shows the average radial profile of PLP-GFP prior to photobleaching (blue line) and 120 s after photobleaching (pink line). Profiles were averaged from 10 separate photobleached centrosomes. Scale bar=1 μm.

We previously used fluorescence recovery after photobleaching (FRAP) to show that the PCM-associated fraction of PLP turned over much more rapidly than the centriole-associated fraction (Martinez-Campos et al., 2004). To test whether the PLP fraction associated with specific regions of the outer PCM also turned over rapidly we combined FRAP with 3D-SIM (Conduit et al., 2014b). This analysis confirmed that the PCM-associated fraction of PLP turned over more rapidly than the centriole-associated fraction of PLP, and revealed that the PLP specifically associated with the outer PCM also turned over relatively rapidly (Fig. 2). Thus, unlike Cnn and Spd-2, which are recruited around the mother centriole and then spread outwards (Conduit et al., 2014b), PLP is continuously being recruited to the PCM scaffold from the cytoplasm, and in particular to certain peripheral regions of the PCM scaffold.

### Centrosomal MTs are perturbed in embryos that lack PLP, but these embryos can develop and hatch at near normal rates

**Fig. 3.**
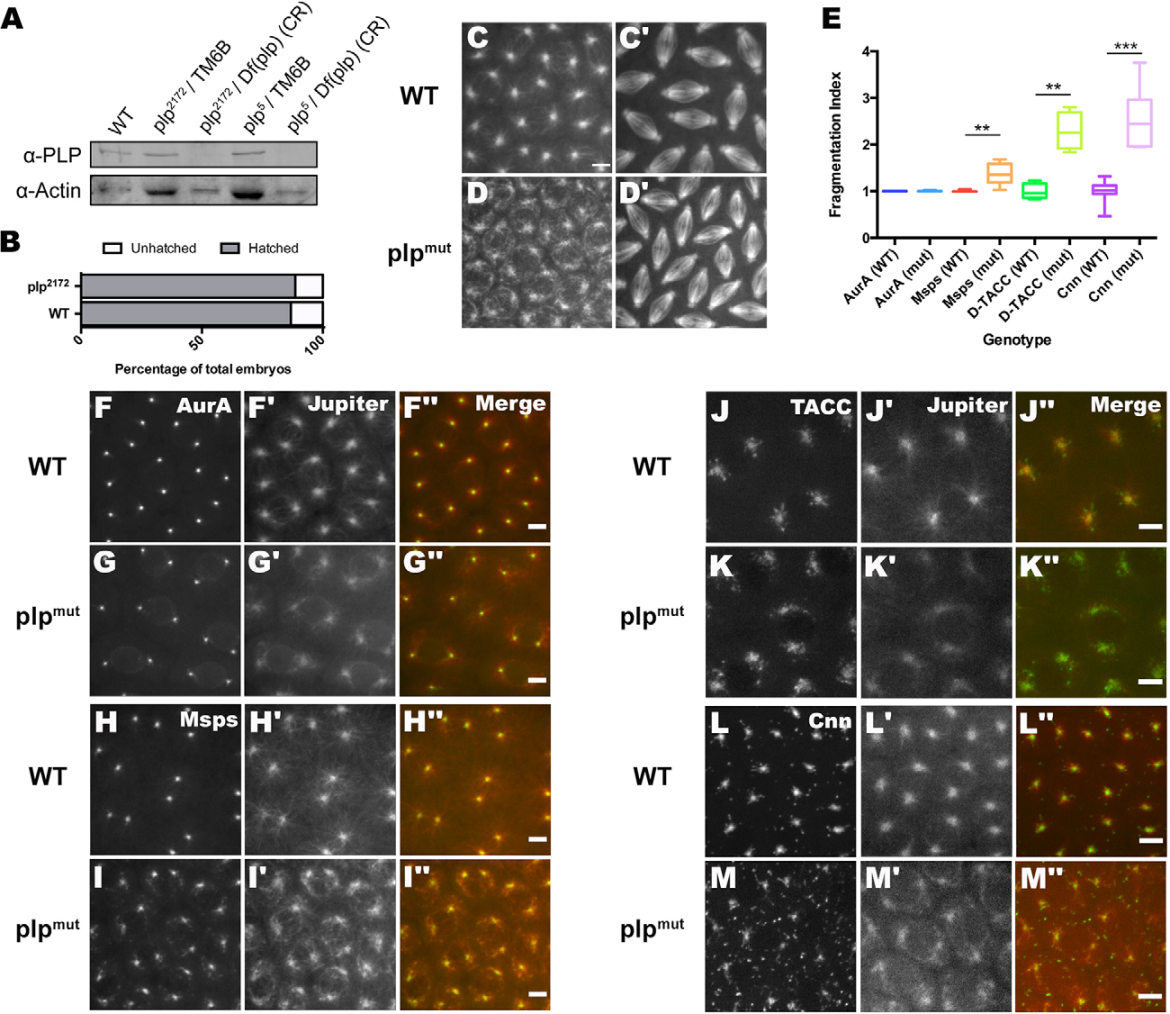
**An analysis of MT and PCM behavior in WT and *plp^mut^* embryos.** (A) A western blot showing PLP levels in WT (lane 1) and *plp^2172^* and *plp^5^* heterozygous (lanes 2 and 4) and homozygous (lanes 3 and 5) mutant embryos. Actin is shown as a loading control. (CR) denotes embryos obtained from “cilia-rescue” flies. Note that approximately double the amount of protein is present in the heterozygous lanes, so the amount of PLP is approximately the same as in WT embryos. PLP is not detectable in the *plp* mutant embryos. (B) Bar graph indicates the percentage of embryos that hatch into 1st instar larvae from cilia-rescue and WT lines. Similar results were obtained with *plp^mut^* germline-clone embryos (data not shown). (C-D′) Images show the localization of the MT-binding Jupiter-mCherry protein in WT and *plp^mut^* embryos. The MTs in S-phase embryos (C,D) are poorly focused around the centrosomes in *plp* mutants, but spindles are relatively well organized by metaphase (C′,D′); see supplementary material Movies S1 and S2. (E) Quantification of the PCM Fragmentation Index of various GFP tagged PCM proteins. Asterisks indicate significant differences between the WT and cilia-rescue lines (two tailed *t*-test: **P≤0.01; ***P≤0.001). Box and whisker plots represent the 25–75th and 0–100th percentile range, respectively. (F-M″) Images show the behavior of various PCM markers (left panels, green in merged images), as indicated, and MTs (middle panels, red in merged images) in WT or *plp^mut^* embryos; see supplementary material Movies S3-S10. Scale bars=5 µm.

We next wanted to analyze the function of PLP in embryos. As PLP is essential for cilia function, homozygous *plp* mutant flies are uncoordinated, are unable to mate or lay any embryos, and die shortly after eclosion (Martinez-Campos et al., 2004). We therefore took two approaches to assess PLP function in early embryos. First, we recombined the *plp^2172^* allele onto an FRT chromosome, allowing us to generate homozygous mutant germ-line clone (GLC) embryos. Second, we established stocks that express full length mCherry-PLP from a UAS-promoter driven by a pan-neuronal elav-Gal4 line in a *plp^2172^* or *plp^5^* mutant background, over a Deficiency chromosome – *Df(plp)* – that completely lacks the *plp* gene (Martinez-Campos et al., 2004). These flies are transheterozygous mutant for *plp* but express mCherry-PLP in their neuronal cells – this rescues the cilia defect in neurons and so these flies are no longer uncoordinated and can mate and lay embryos (see Materials and Methods for full details). We term these “cilia rescue” flies. Western blot analysis confirmed that embryos produced by either method lacked detectable PLP (Fig. 3A; data not shown), and both sets of embryos exhibited the same phenotypes that we describe below; for ease of presentation we simply refer to these embryos collectively as *plp* mutants (*plp^mut^*).

To our surprise, *plp^mut^* embryos developed and hatched at near normal rates (Fig. 3B), demonstrating that, unlike proteins such as Cnn, Spd-2, Asl and TACC (that are not essential for fly viability, but are essential for early embryo development), PLP is not essential for mitosis even during the very rapid nuclear divisions of the syncytial embryo. We assessed MT behavior in living *plp^mut^* embryos expressing either RFP-tubulin or the MT binding protein Jupiter-mCherry, and found that the centrosomal MTs were consistently more diffuse and disorganized than in wild-type (WT) controls during S-phase – when many small aggregates of MTs would continuously break away from the periphery of the centrosomes (Fig. 3C,D; supplementary material Movies S1 and S2) – but were largely unperturbed by the time spindles were fully formed in metaphase (Fig. 3C′,D′; supplementary material Movies S1 and S2). Thus, as previously reported in brain cells (Martinez-Campos et al., 2004), *plp^mut^* embryos show subtle MT abnormalities early in mitosis, but are largely normal by metaphase.

### The outer region of the PCM is abnormally dispersed in the absence of PLP

We wondered whether the MT defects we observed in *plp^mut^* embryos might be caused by defects in the organization of the outer PCM (where PLP appears to be concentrated – Fig. 1). To test this possibility, we expressed GFP-fusions to centrosomal components that were concentrated in the inner region of the PCM (Aurora A-GFP) an intermediate region of the PCM (Msps-GFP) and to the outer most regions of PCM (GFP-TACC and GFP-Cnn) (Fig. 3F-M; supplementary material Movies S3-S10). The centrosomal localization of these proteins showed a graded perturbation in the absence of PLP. A small number of very small flares were observed for Aurora A-GFP in the *plp* mutants, but not in WT controls (Fig. 3F,G; supplementary material Movies S3 and S4). Msps-GFP exhibited a stronger phenotype and could be seen “flaring” excessively from the periphery of the centrosome in the *plp* mutants (Fig. 3H,I; supplementary material Movies S5 and S6). The outer PCM components GFP-TACC and GFP-Cnn exhibited the strongest defects; the proteins were still concentrated at centrosomes, but they exhibited a much more fragmented appearance in the outer regions, forming many more flares than in WT embryos that moved rapidly away from the centrosomes on the centrosomal MTs (Fig. 3J-M; supplementary material Movies S7-S10).

We quantified these defects in PCM organization by calculating a “fragmentation index” (FI), based on the number of discrete particles of each GFP-fusion that could be detected concentrated around the centrosomes in WT (normalized to 1) and *plp^mut^* embryos (see Materials and Methods). The FIs of GFP-TACC, GFP-Cnn and Msps-GFP (but not Aurora-A-GFP) were dramatically and significantly increased in *plp^mut^* embryos (Fig. 3E). We conclude that the PCM in the outer regions of the centrosome is abnormally dispersed in the absence of PLP, while the distribution of more inner centrosomal proteins, like Aurora A, appears largely unperturbed. The extensive dispersal of the outer PCM made it difficult to accurately quantify centrosome size in *plp^mut^* embryos.

### Y2H analysis of PLP identifies several potential self-interactions and interactions with Asl, Spd-2 and Cnn

**Fig. 4.**
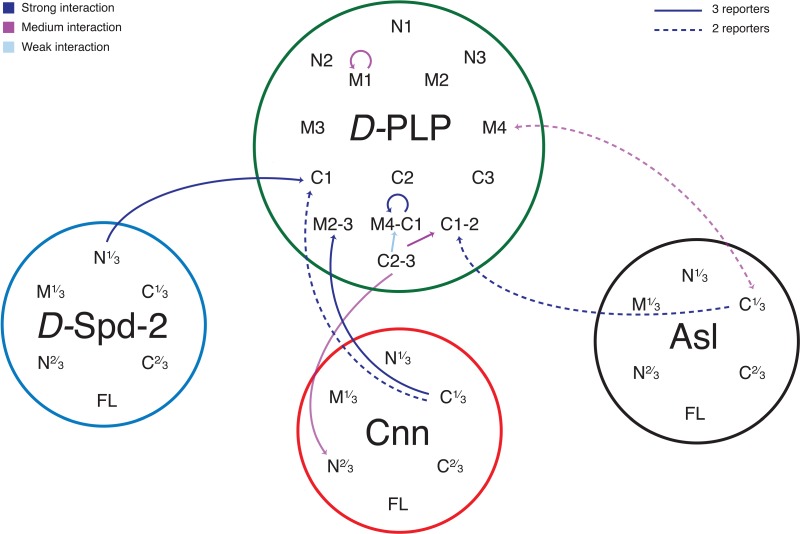
**A schematic summary of the interactions identified by yeast two-hybrid (Y2H) between PLP, Asl, Cnn and Spd2.** The diagram illustrates the interacting regions identified by Y2H: solid lines indicate interactions where all three reporter genes tested were activated, heavy dashed lines where two reporters were activated. The color of the line indicates the strength of the interaction (estimated by the amount of colony growth), as indicated. Arrowheads point towards the prey protein, and double-headed arrows indicate that the interaction was seen with both proteins as bait and prey; see supplementary material Table S1 for the full dataset.

To identify interactions that may be important for PLP's role in maintaining the integrity of the outer PCM we performed a yeast two-hybrid (Y2H) screen between fragments of PLP and fragments of the three proteins most closely associated with mitotic PCM assembly in early embryos – Asl, Spd-2 and Cnn (Conduit and Raff, 2010; Conduit et al., 2014b) (see Materials and Methods for details). These results identified several interactions with all three proteins as well as several self-interactions (Summarized in Fig. 4; see supplementary material Table S1 for full details of all interactions tested).

**Fig. 5.**
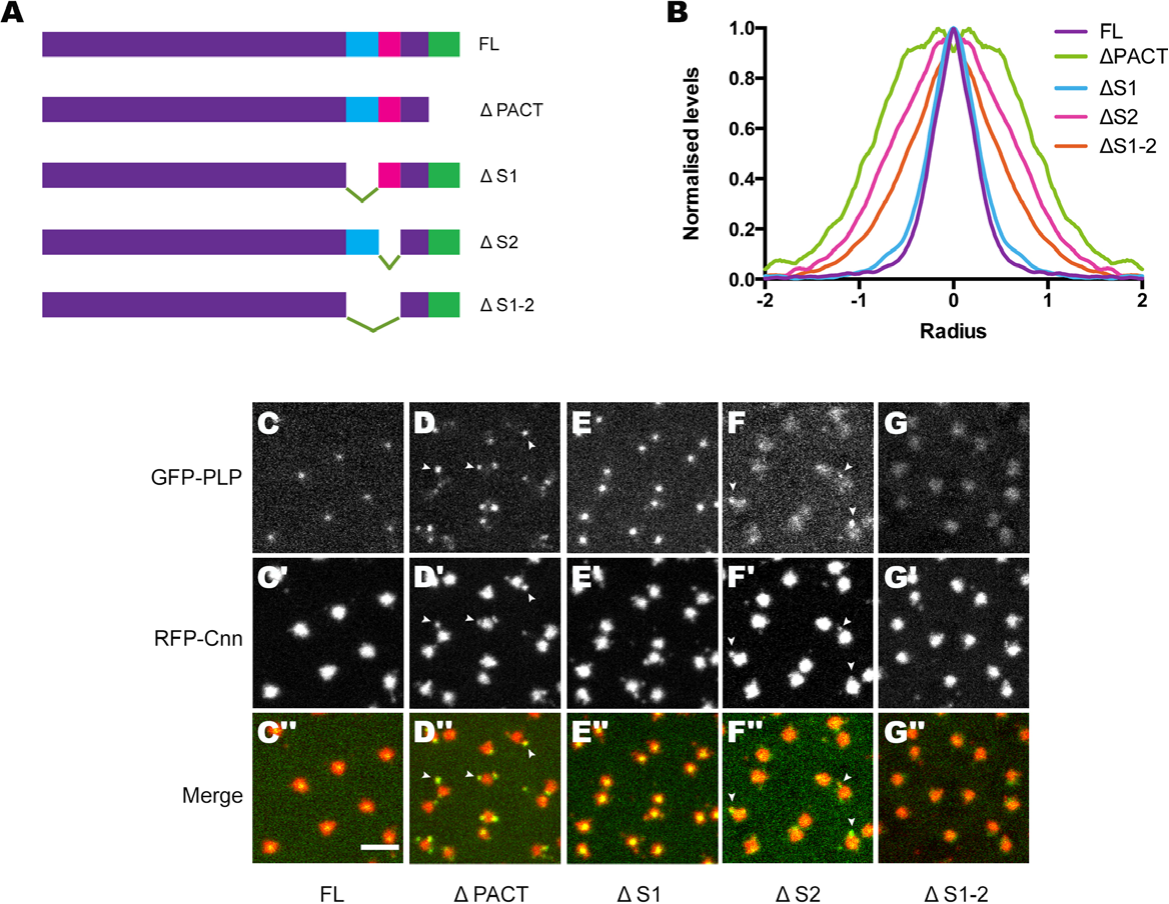
**An analysis of the localization of various deleted forms of PLP in WT embryos.** (A) A schematic illustration of the different PLP deletion constructs. (B) The graph shows the average radial profile of the various deletion constructs at centrosomes; radius is measured in µm. The deletion constructs were injected into WT embryos expressing RFP-Cnn or Asl-mCherry; the Cnn or Asl signal was used to calculate the center of mass of the centrosome; the distribution of the PLP deletion proteins around the center was then measured by radial profiling (see Materials and Methods). (C-G″) Images show the localization of the various GFP-PLP deletion constructs (top panels, green in merged images), as indicated below the panels, in WT embryos expressing RFP-Cnn (middle panels, red in merged images). Arrowheads in (D-D″) indicate the co-localization of GFP-PLP-ΔPACT and RFP-Cnn in the Cnn flares. Scale bar=4 µm.

Using these Y2H interacting regions as a starting point, we performed a secondary structure prediction analysis to identify potential structural features within each region, and we also performed multiple sequence alignments to identify conserved regions. We then designed several deletion constructs that we reasoned would disrupt structurally distinct regions of PLP that were likely to be involved in these interactions, without disrupting the overall folding of the PLP protein: PLP-ΔPACT (deleting aa 2672–2897), which removes the previously characterized centriole targeting PACT-domain of PLP (Gillingham and Munro, 2000); PLP-ΔS1 (deleting aa 2123–2322), which removes most of the region of PLP that interacted strongly with Asl in the Y2H analysis; PLP-ΔS2 (deleting aa 2330–2471), which removes a region of PLP that showed strong Y2H interactions with Spd-2, Cnn and Asl, as well as strong self-interactions; and PLP-ΔS1-2 (deleting aa 2123–2471), which combines the ΔS1 and ΔS2 deletions (Fig. 5A).

Each of the deletions (as well as the full length cDNA) were cloned as GFP fusions and transcribed *in vitro* to make GFP-PLP mRNA that was then injected into WT embryos (that contain endogenous WT PLP) that expressed either RFP-Cnn or Asl-mCherry. Embryos were imaged 60–120 min later and the distribution of each GFP-fusion protein at the centrosome was analyzed visually (Fig. 5C-G) and quantitatively using radial profiling to measure the spread of the proteins around the centriole (Conduit et al., 2014a) (see Materials and Methods; Fig. 5B).

The full-length construct (PLP-FL) localized strongly to centrioles and it was also weakly detectable in the PCM (Fig. 5C). As expected, PLP-ΔPACT was unable to localize to the centriole as it was missing its previously characterized centriole targeting domain; interestingly, however, PLP-ΔPACT localized very strongly to the peripheral regions of the PCM, where it almost always colocalized with Cnn flares (Fig. 5D, arrowheads), and this concentration in the outer regions was reflected in its much broader radial profile (Fig. 5B). This finding suggests that PLP can be targeted to these peripheral regions of the PCM independently of the PACT domain, and that this targeting is stronger for PLP molecules that lack the PACT domain and cannot be recruited to centrioles. PLP-ΔS1 exhibited a very similar localization to the full-length protein, although its tight distribution around the centriole was slightly broader (Fig. 5B,E), while PLP-ΔS2 was more prominent in the PCM than in the centriole, and was also enriched to some extent in the Cnn flares (Fig. 5B,F). PLP-ΔS1-2 was also prominently localized to the PCM, rather than the centriole, but it was no longer detectably enriched in the peripheral Cnn flares, suggesting that the S1-2 region is required to target PLP specifically to the outer regions of the PCM (Fig. 5B,G). These findings demonstrate that multiple regions of PLP are required to ensure its proper localization, and that there is a complex interplay between these regions, which can lead to several different localization patterns when one or more regions of PLP is deleted. Moreover, as described below, the recruitment of these deletion constructs to the various regions of the PCM is at least partially dependent upon the presence of endogenous full length PLP.

### The interactions of PLP through the S2 region are essential for its role in maintaining the outer PCM

**Fig. 6.**
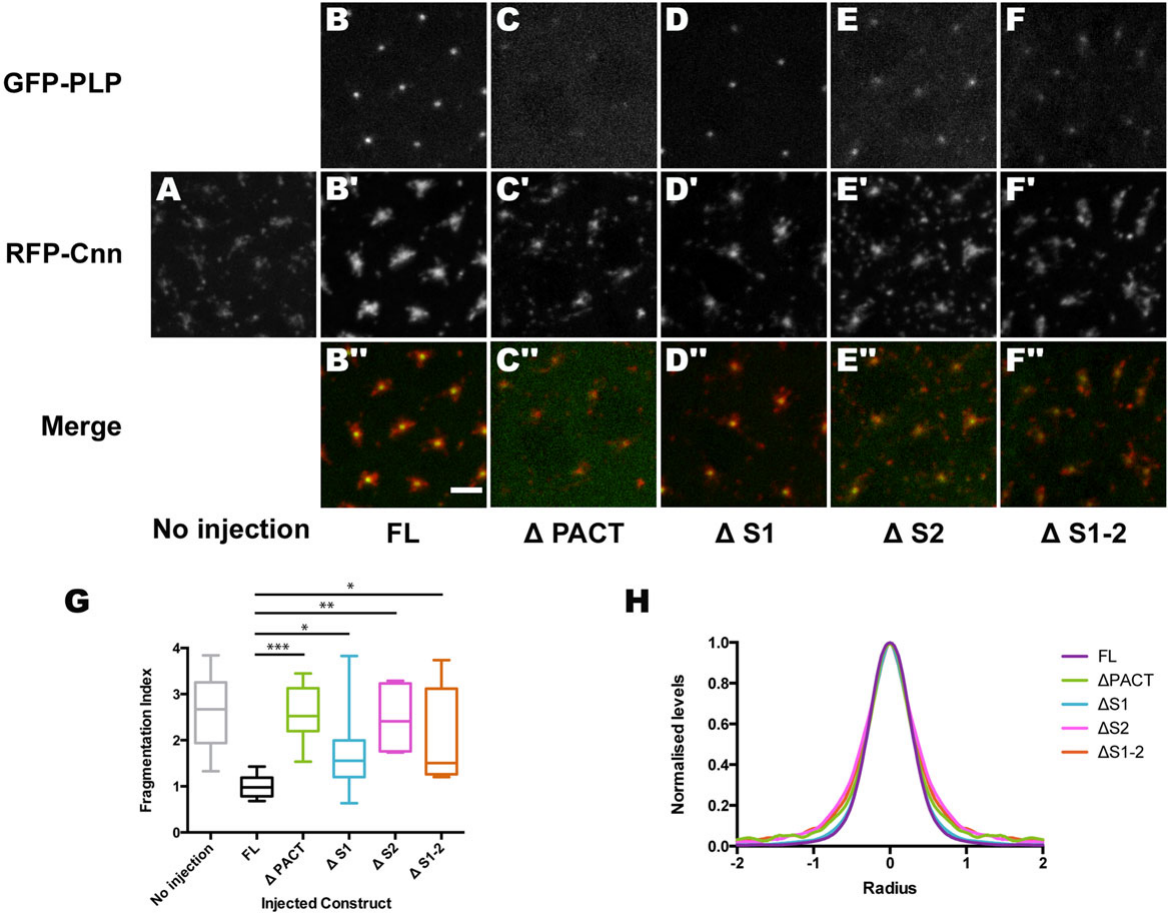
**An analysis of the localization of various deleted forms of PLP in *plp^mut^* embryos.** (A) Image shows the distribution of RFP-Cnn in a *plp^mut^* embryo. (B-F″) Images show the distribution of RFP-Cnn (middle panels, red in merged images) in *plp^mut^* embryos injected with mRNA encoding the various GFP-PLP deletion constructs (top panels, green in merged images), as indicated below the panels. The deleted proteins rescue the PCM fragmentation of the Cnn to varying degrees. (G) Quantification of the “rescuing” of the RFP-Cnn Fragmentation Index in the presence of the various fusion proteins; asterisks indicate significant differences (two tailed *t*-test: *P≤0.05; **P≤0.01; ***P≤0.001). Box and whisker plots represent the 25–75th and 0–100th percentile range, respectively. (H) The graph shows the average radial profile of the various deletion constructs, as indicated, at centrosomes; radius is measured in µm. The deletion constructs were injected into *plp^mut^* embryos expressing RFP-Cnn. The Cnn signal was used to calculate the center of mass of the centrosome; the distribution of the PLP deletion proteins around the center was measured by radial profiling. Scale bar=4 µm.

We next wanted to test the ability of the various GFP-PLP deletions to rescue the PCM fragmentation phenotype observed in *plp^mut^* embryos. We therefore injected mRNAs encoding the various deletion constructs into *plp^mut^* embryos expressing RFP-Cnn and calculated the fragmentation index of the RFP-Cnn 60–120 min later (Fig. 6G). PLP-FL strongly rescued the PCM fragmentation defect when compared to un-injected controls (Fig. 6A,B,G). PLP-ΔPACT showed no rescue activity (Fig. 6C,G), which was surprising as in WT embryos (that contained endogenous WT PLP) this construct strongly localized to the outer Cnn flares. In the *plp^mut^* embryos, however, the PLP-ΔPACT no longer localized strongly to the Cnn flares, and its localization to the centrosomal region in general was extremely weak. Thus, the recruitment of the PLP-ΔPACT to the PCM we observed in WT embryos (Fig. 5D) appears to require WT endogenous protein containing a PACT-domain. In the absence of endogenous WT PLP, only very small amounts of PLP-ΔPACT can be recruited to centrosomes and this cannot detectably rescue the PCM fragmentation phenotype (Fig. 6G).

As in WT embryos, PLP-ΔS1 localized strongly to centrioles in *plp^mut^* embryos and it rescued the PCM fragmentation defect, although not as well as PLP-FL (Fig. 6D,G). In contrast PLP-ΔS2 and PLP-ΔS1-2 both only localized weakly to the centrosomal region; this localization was not as weak as that observed with PLP-ΔPACT, and both proteins appeared to still concentrate at centrioles and in the PCM (Fig. 6E-G). Their localization in the PCM was not as strong as that observed in the presence of WT endogenous PLP (Fig. 5F,G), again suggesting that interactions with the endogenous protein play an important part in the recruitment of these proteins to the PCM in WT embryos. Interestingly, however, although significant amounts of both proteins were still recruited to centrosomes, neither protein was able to properly rescue the PCM fragmentation defect (Fig. 6G). Taken together, these data strongly suggest that the S2 region (that can interact with Asl, Spd2 and Cnn in Y2H assays) and the PACT domain are both required to properly stabilize the outer regions of the PCM.

In summary, we have shown that PLP is not essential for mitosis even in the rapidly dividing early embryo. Nevertheless, PLP is recruited to the PCM, and, in particular, to specific areas of the outermost region of the Cnn scaffold that extends away from the centrioles along the centrosomal MTs. PLP seems to play an important part in strengthening the outer regions of the PCM, and our Y2H assays and deletion construct analysis indicate that PLP exhibits a complex network of potential self interactions and potential heterologous interactions with several other key PCM proteins, including Cnn, to fulfill this role. Thus, as appears to be the case in several other systems (Buchman et al., 2010; Graser et al., 2007; Haren et al., 2009; Kim and Rhee, 2014; Wang et al., 2010), the interaction between PLP and Cnn also has an important role in mitotic PCM assembly in flies, although, unlike Cnn, PLP is not essential for the viability of the early embryo. We speculate that PLP may form important interactions with several PCM components, thus helping to strengthen the structure of particularly the outer PCM region in fly embryos. Clearly it will be important to determine how the absence of PLP influences the dynamics of the recruitment and retention of the various components of the PCM.

## MATERIALS AND METHODS

### *Drosophila* lines

UASg-mCherry-PLP flies were made by introducing a full-length D-PLP cDNA (Kawaguchi and Zheng, 2004) into the pUASg-mCherry-attB vector, modified from pUASg-attB (Bischof et al., 2007) by addition of mCherry into the AgeI restriction site. Transgenic lines were generated by injection into the y[1] M{vas-int.Dm}ZH-2A w[*]; M{3xP3-RFP.attP'}ZH-51C landing site (Bischof et al., 2007) by the Fly Facility in the Department of Genetics, Cambridge, UK. Other GFP, RFP, and mCherry fusions have been described previously: PLP-GFP (Conduit et al., 2014b), GFP-Cnn and Aur-A-GFP (Lucas and Raff, 2007), RFP-Cnn (Conduit et al., 2010), Jupiter-mCherry (Callan et al., 2010), Msps-GFP (Lee et al., 2001) and TACC-GFP (Gergely et al., 2000). The *Df(3L)Brd15* deficiency [referred to as *Df*(*plp*)] and the P-element insertion line *l(3)s2172* (referred to as *plp^2172^*) were obtained from Bloomington, while the strong EMS allele *plp^5^* was described previously (Martinez-Campos et al., 2004).

To analyze the role of PLP in early embryos we initially used standard methods to recombine the *plp^2172^* mutation on to an *FRT80* chromosome and this stock was used in combination with an *FRT80, OvoD* stock to produce germline-clone embryos that lacked the maternal contribution of PLP (Chou and Perrimon, 1996). We also constructed the following “cilia rescue” lines that are mutant for *plp*, but express UASg-mCherry-PLP only in the nervous system of the fly, thus rescuing the uncoordinated phenotype of *plp* mutants:

*w^67^*; UASg-mCherry-PLP/elavGal4; *plp^2172^*, Msps-GFP/*Df(plp)*, Jupiter-mCherry

*w^67^*; UASg-mCherry-PLP/elavGal4; *plp^2172^*, TACC-GFP/*Df(plp)*, Jupiter-mCherry

*w^67^*; UASg-mCherry-PLP/elavGal4; *plp^2172^*, Aur-A-GFP/*Df(plp)*, Jupiter-mCherry

*w^67^*; UASg-mCherry-PLP/elavGal4; *plp^5^*, GFP-Cnn/*Df(plp)*, Jupiter-mCherry

PLP deletion constructs were analyzed in the WT background by injecting mRNA into either RFP-Cnn, or Asl-mCherry embryos.

To analyze the PLP deletion constructs in the *plp^2172^* background the following line was constructed: w^67^; UASg-mCherry-PLP/elavGal4 ; *plp^2172^*, RFP-Cnn/*Df(plp)*.

### Antibodies

For immunofluorescence analysis we used the following antibodies: sheep anti-Cnn (1:500) (Cottee et al., 2013); rabbit anti-PLP (PPHA) (1:500) (Martinez-Campos et al., 2004); mouse anti-TACC (1:500) (Gergely et al., 2000); rat anti-Asl (1:500) and rat anti-Spd2 (1:500) (Baumbach et al., 2015). Secondary antibodies were from Molecular Probes (Life Technologies): Alexa Fluor 405, 488 and 592 (all used at 1:1000). To enhance GFP fluorescence for 3D-SIM on fixed embryos we used GFP-Booster ATTO488 1:500 (Chromotek). For western blotting rabbit anti-PLP (PPHA) (1:1000) (Martinez-Campos et al., 2004) and mouse anti-actin (1:1000) (Sigma) were used. Secondary antibodies conjugated to IRDye 680 and IRDye800 were used at a concentration of 1:10,000 and blots imaged on an Odyssey CLx imager (LI-COR).

### Yeast two-hybrid

Bait and prey fragments were cloned, introduced into yeast, and tested for interactions as described previously (Conduit et al., 2014a). For the baits (except PLP), fragments encoding the N-terminal, middle, and C-terminal thirds of the proteins were cloned, along with fragments encoding the N-terminal two- thirds, C-terminal two-thirds, and the full-length protein. For the preys (except PLP), smaller ∼200 aa fragments and larger combinations of these fragments, including the full-length protein, were cloned. As PLP is such a large protein for the baits it was cloned as fragments of ∼300 aa, and larger combinations up to ∼1000 aa. For PLP prey constructs fragments of ∼200 aa and larger combinations up to ∼1000 aa were cloned.

### PLP deletions and RNA injection assays

Using the interacting regions identified by Y2H as a starting point we designed deletions of PLP that we reasoned would remove potential functional domains, but would not disrupt overall protein folding. We performed secondary structure analysis with Coils (Lupas et al., 1991) and PSIPRED (Buchan et al., 2013), and we identified regions of sequence conservation by performing multiple sequence alignments with Clustal W2 (Larkin et al., 2007); appropriate deletions were designed that removed conserved structural regions. PLP deletions were generated by site directed mutagenesis of pDonr-Zeo-PLP using the Q5 site directed mutagenesis kit (NEB). Deletions were cloned via Gateway (Invitrogen) into the pRNA-GFP destination vector (Conduit et al., 2014a). mRNA was synthesized and purified as described previously (Cottee et al., 2015).

### Radial profiles of GFP-PLP deletion constructs in embryos

Radial profiling was performed as described (Conduit et al., 2014b) using ImageJ. Radial profiles were calculated for maximal intensity projections of fluorescence in order to calculate the spread of the various PLP deletion constructs around the centriole. Either Asl-mCherry or RFP-Cnn signal was used to calculate the center of mass of the centrosome and determine the location of the centriole.

### PCM fragmentation analysis

Fragmentation analysis was performed on maximal intensity projections of fluorescence using ImageJ. A manually drawn mask was applied to the image around the nuclei of interest. The number of centrosomes was then calculated by performing a gaussian blur, removal of noise, and finding maxima in the Jupiter-mCherry channel (when measuring fragmentation of GFP tagged PCM components) or the GFP-PLP channel in the case of mRNA injection into RFP-Cnn embryos (to measure rescue of Cnn fragmentation). In cases where the GFP-PLP signal was too diffuse (such as with the ΔPACT construct) centrosomes were counted manually. Noise removal was performed on the channel of interest (GFP tagged PCM component, or RFP-Cnn) and the maxima counted. The fragmentation index was expressed as the number of PCM fragments per centrosome. For easier graphical representation this was then normalized to the control sample in each pair. At least ten centrosomes per embryo and at least five embryos were analyzed for each sample.

### Western blotting

Embryos were fixed in methanol, rehydrated in PBS, sorted by age and boiled in sample buffer. Samples containing 20 embryos were run on NuPAGE 3–8% Tris-acetate pre-cast gels (Life Technologies). The proteins were transferred onto Immobilon-FL membrane and loading was initially checked using Ponceau staining. The membrane was then blocked with 3% Milk powder, 1% BSA, and probed with antibodies against PLP, and against actin as a loading control.

### Live imaging of *Drosophila* embryos

Syncytial stage embryos were imaged on a Perkin Elmer ERS Spinning Disk confocal system (ERS software) mounted on a Zeiss Axiovert microscope, using a 63× 1.4 NA oil-immersion objective. Alternatively embryos were imaged on a Perkin Elmer Ultra-VIEW VoX (Volocity software) mounted on an IX81 microscope (Olympus), using a 60× 1.3 NA silicon immersion objective and an electron-multiplying charge-coupled device camera (ImagEM, Hamamatsu Photonics). All control/experiment pairs were analyzed on the same microscope system with identical illumination and acquisition settings.

For experiments to analyze the effects of PLP deletions, mRNAs encoding GFP-PLP deletions were injected into embryos and then imaged 60–120 min later.

### Super-resolution 3D structured illumination microscopy

3D-SIM microscopy was performed as described in (Conduit et al., 2014b) on a DeltaVision OMX V3 Blaze microscope (GE Healthcare, UK). Images shown are maximum intensity projections of several z-slices. 3D-SIM microscopy was performed on both live and fixed *Drosophila* embryos. The radial profiles shown for the 3D-SIM FRAP experiment spanned 3.26 μm with a concentric ring every 0.00816 μm.

## Supplementary Material

Supplementary Material
