# Supplementary material for: The Drosophila Pericentrin-like-protein (PLP) cooperates with Cnn to maintain the integrity of the outer PCM
Source: Biol Open. 2015 Jul 8;4(8):1052–61. doi: 10.1242/bio.012914 (PMC4542290; doi:10.1242/bio.012914)
Supplement: Supplementary Material [file supp_4_8_1052__index.html]

The Drosophila Pericentrin-like-protein (PLP) cooperates with Cnn to maintain the integrity of the outer PCM — The Drosophila Pericentrin-like-protein (PLP) cooperates with Cnn to maintain the integrity of the outer PCM — Supplementary Material 

# The *Drosophila* Pericentrin-like-protein (PLP) cooperates with Cnn to maintain the integrity of the outer PCM

## BIO012914 Supplementary Material

- Supplementary Material
