# Supplementary material for: The Drosophila Pericentrin-like-protein (PLP) cooperates with Cnn to maintain the integrity of the outer PCM
Source: Biol Open. 2015 Jul 8;4(8):1052–61. doi: 10.1242/bio.012914 (PMC4542290; doi:10.1242/bio.012914)
Supplement: Supplementary Material [file supp_bio.012914_BIO012914supp.pdf]

Supplementary materials

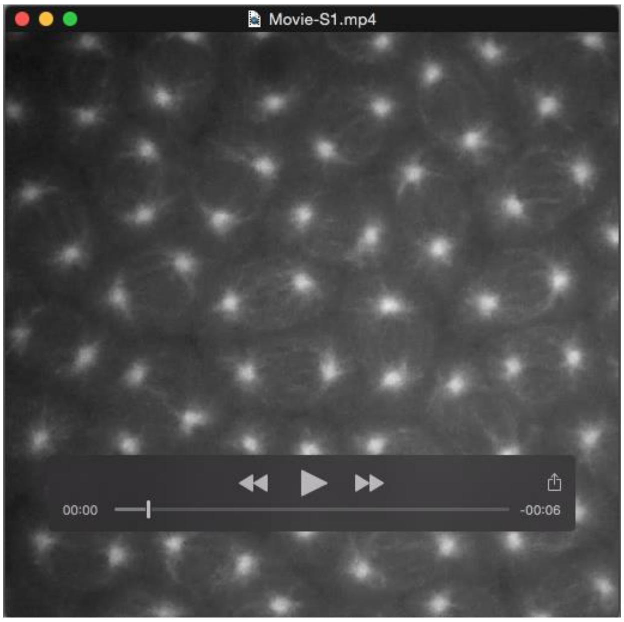

Movie S1

A WT embryo expressing Jupiter-mCherry to label the MTs.

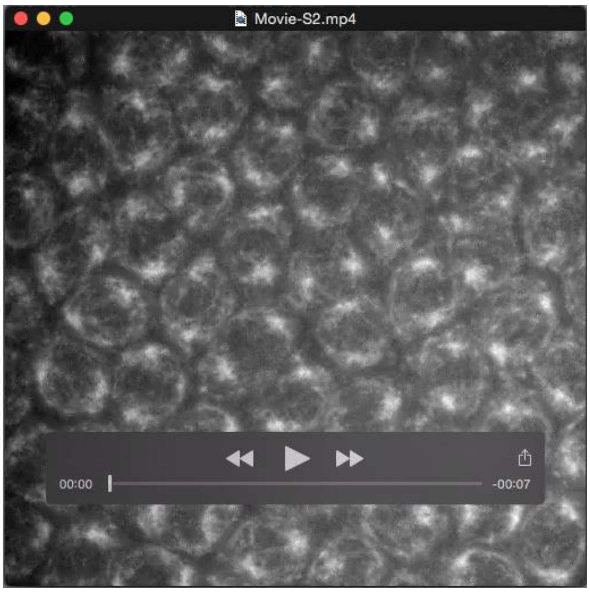

Movie S2

A *plp*^{mut} embryo expressing Jupiter-mCherry to label the MTs.

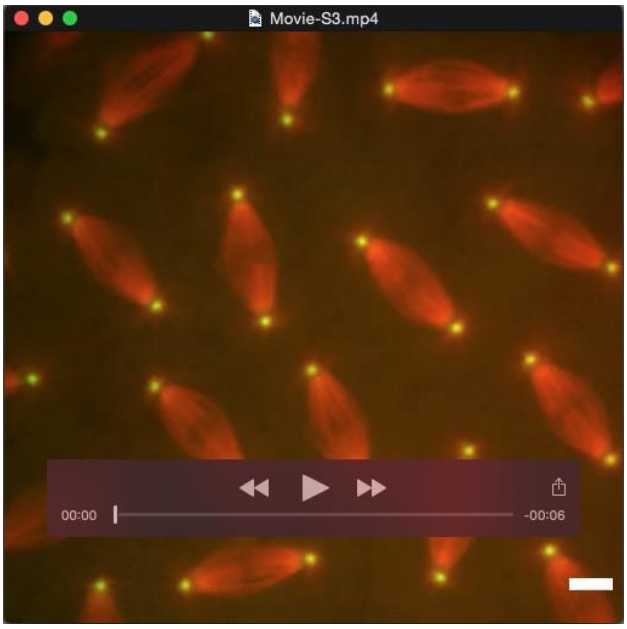

Movie S3

A WT embryo expressing Jupiter-mCherry (red) and Aurora A-GFP (green).

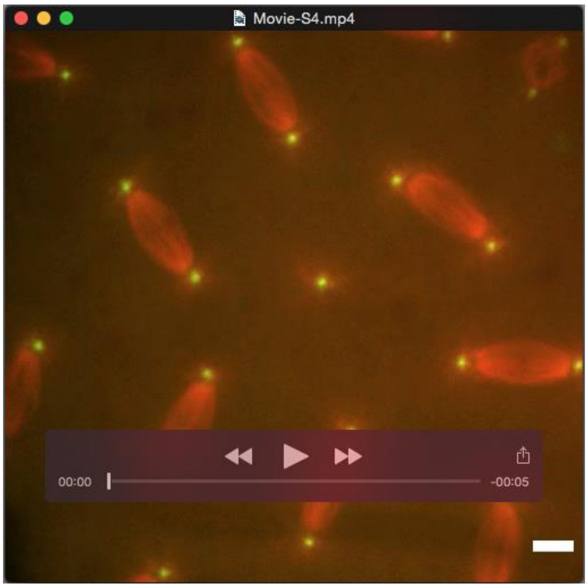

Movie S4

A *plp*^{mut} embryo expressing Jupiter-mCherry (red) and Aurora A-GFP (green).

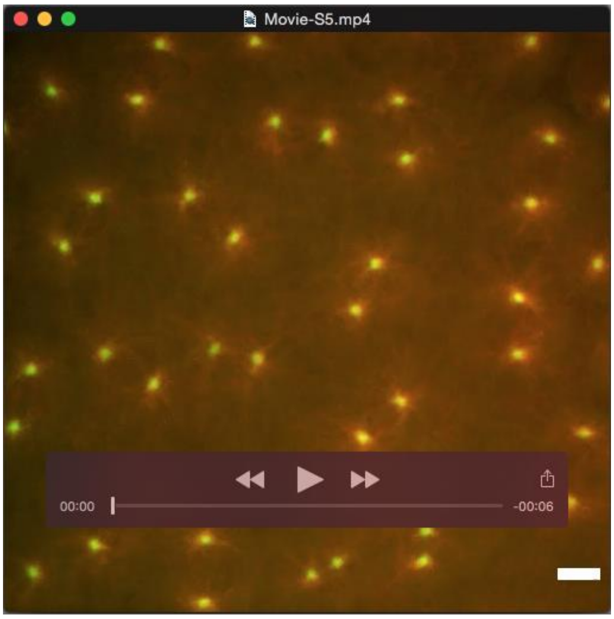

Movie S5

A WT embryo expressing Jupiter-mCherry (red) and Msps-GFP (green).

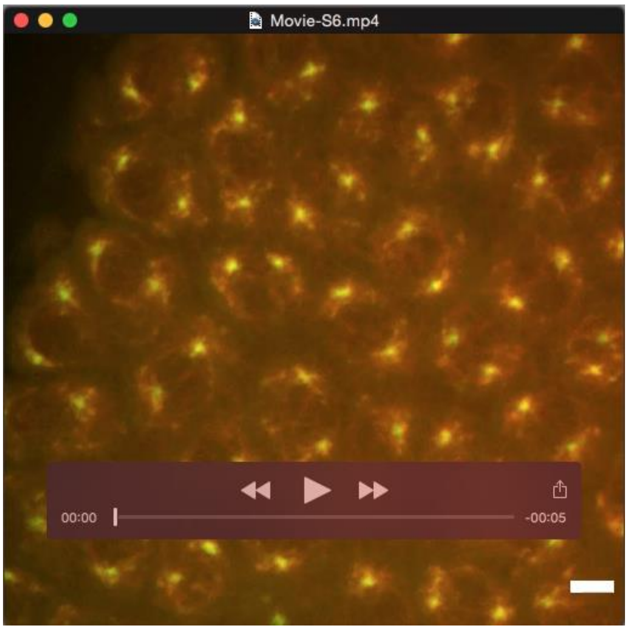

Movie S6

A *plp*^{mut} embryo expressing Jupiter-mCherry (red) and Msps-GFP (green).

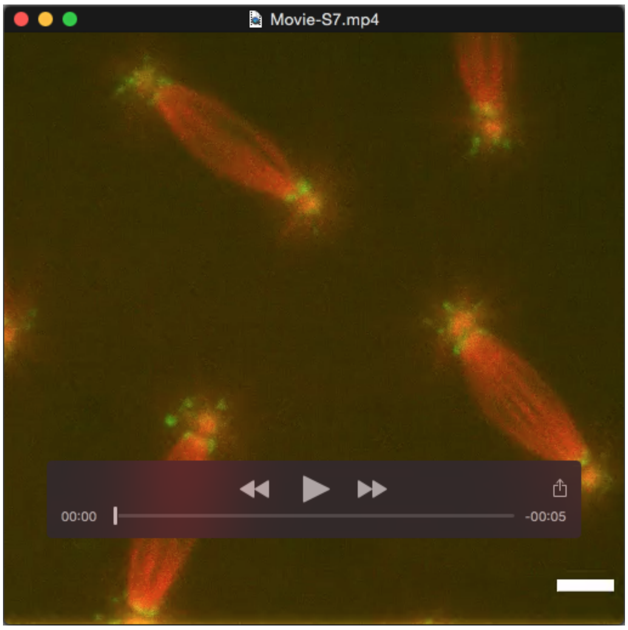

Movie S7

A WT embryo expressing Jupiter-mCherry (red) and GFP-TACC (green).

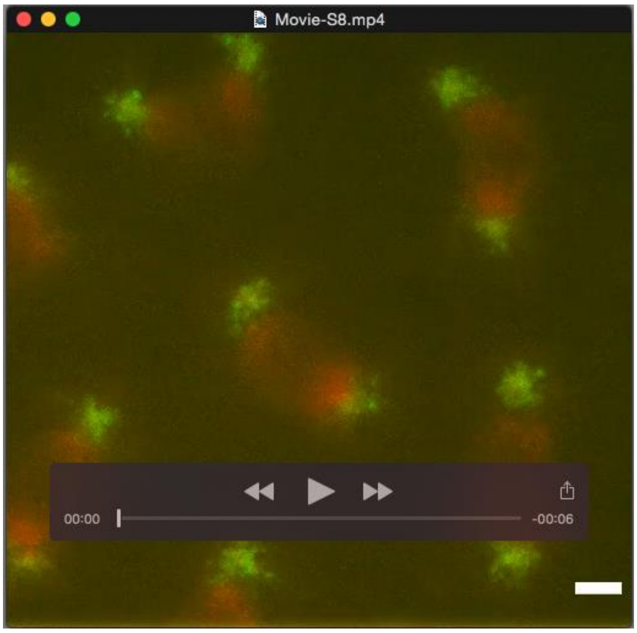

Movie S8

A *plp*^{mut} embryo expressing Jupiter-mCherry (red) and GFP-TACC (green).

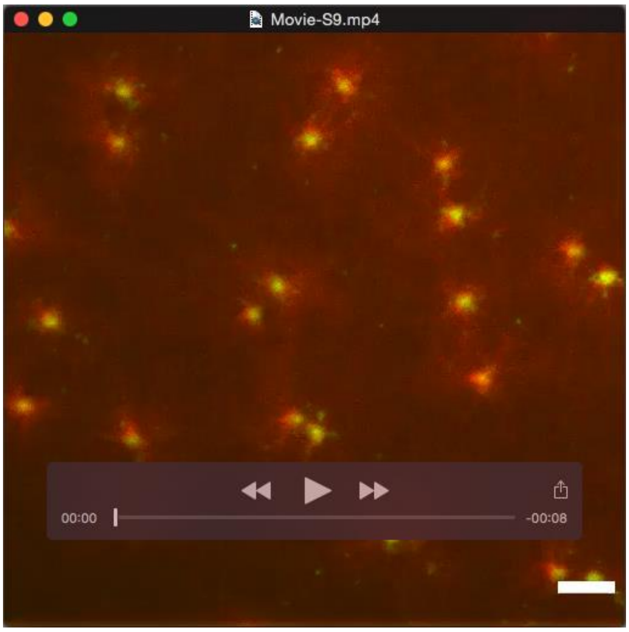

Movie S9

A WT embryo expressing Jupiter-mCherry (red) and GFP-Cnn (green).

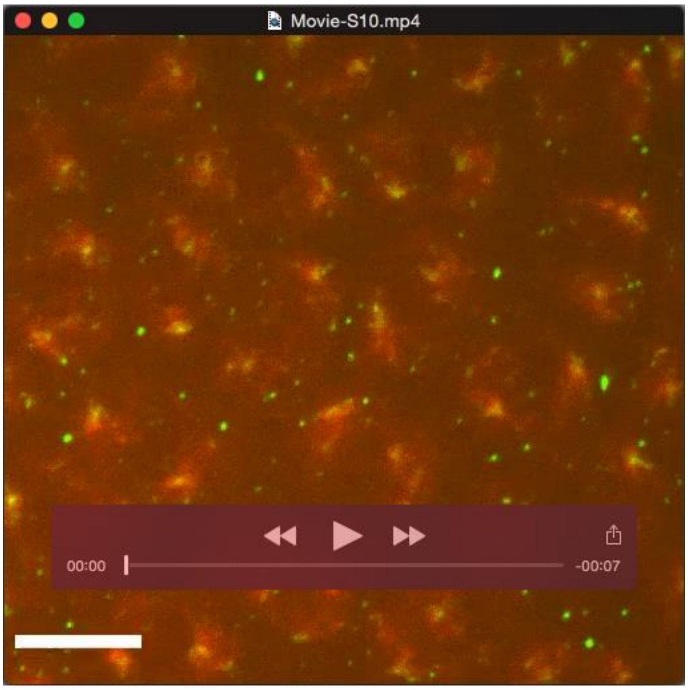

Movie S10

A *plp*^{mut} embryo expressing Jupiter-mCherry (red) and GFP-Cnn (green).

Table S1. A schematic summary of the interactions identified by Yeast Two-Hybrid (Y2H) between PLP, Asl, Cnn and Spd2. Related to Fig. 4. Yeast-2-hybrid analysis was carried out using various bait and prey fragments of each protein (as indicated in columns A and B). Three different reporters were tested – His (columns C-E), Ade (columns F,G), and LacZ (column H). Interaction levels are indicated as strong, medium, weak or none. PLP baits and preys were tested against all Asl, Cnn and Spd2 fragments, but only those that scored positive in at least one assay are shown. Amino acid residues fragments and their sizes are indicated in columns I-N.

Click here to Download Table S1
